# Dietary α-Tocopherol Deficiency Disrupts Hepatic Circadian Clock and Lipid Metabolism in Association with Gut Microbiota Dysbiosis

**DOI:** 10.3390/nu18121853

**Published:** 2026-06-09

**Authors:** Lei Peng, Yan Zhao, Yuqin Fan, Qi Peng, Jun Sheng, Yang Tian, Xiaoyu Gao

**Affiliations:** 1 Yunnan Key Laboratory of Precision Nutrition and Personalized Food Manufacturing, Yunnan Agricultural University, Kunming 650201, China; 2015042@ynau.edu.cn (L.P.); 2024240164@stu.ynau.edu.cn (Y.F.); 2025210142@stu.ynau.edu.cn (Q.P.); 2 Engineering Research Center of Development and Utilization of Food and Drug Homologous Resources, Ministry of Education, Yunnan Agricultural University, Kunming 650201, China; 3 College of Food Science and Technology, Yunnan Agricultural University, Kunming 650201, China; shengj@ynau.edu.cn; 4 Division of Science and Technology, Yunnan Agricultural University, Kunming 650201, China; 2021013@ynau.edu.cn

**Keywords:** circadian clock, alpha-tocopherol, lipid metabolism, *Alistipes*, vitamin, Bmal1, clock, Per2, Scd1

## Abstract

**Background/Objectives** As a fat-soluble vitamin, vitamin E (VE) is prone to suboptimal intake in the general population. Alpha-tocopherol (α-TE) represents the most biologically significant form of VE in vivo. Nevertheless, the potential detrimental effects of α-TE deficiency on health remain unclear. This study was conducted to investigate the effect of α-TE deficiency on hepatic metabolism and gut microbiota. **Methods** C57BL/6J mice were randomly assigned to receive one of three dietary regimens: a α-TE-deficient diet, a control diet with normal α-TE, or a high-dose diet containing four times the normal α-TE level. Histopathology, serum biochemistry, RNA-Seq, RT-qPCR, Western blot, and 16S rRNA gene sequencing with correlation analysis were used to assess metabolic phenotypes, hepatic circadian, hepatic lipid metabolism, and cecal microbiota, respectively. **Results** The results demonstrated that α-TE deficiency induced hepatic steatosis and lipid metabolic disturbances. α-TE deficiency significantly decreased Arntl and Clock expression, but increased Per2. Additionally, it upregulated the expression of lipogenic genes such as *Scd1*, *Elovl6,* and *Elovl3* and simultaneously downregulated fatty acid oxidation genes such as *Cyp4a10*, *Cyp4a14,* and *Acot1*, bringing about imbalance in lipid homeostasis. In addition, α-TE deficiency greatly changed the structure and composition of gut microbiota. Bacterial genera like *Alistipes*, *norank_f__Muribaculaceae*, *Muribaculum*, *Odoribacter*, and *Dubosiella* were significantly correlated with hepatic circadian and lipid metabolism gene expression with the strongest correlation being *Alistipes*. **Conclusions** This work is the first to reveal that short term α-TE deficiency could cause lipid metabolic disorder via the “gut microbiota–liver circadian clock” axis, which provides novel insights into the etiology of nutrition-related metabolic diseases and targets for nutritional intervention.

## 1. Introduction

Circadian rhythm is the internal timing system that is present in all organisms and is driven by a transcription translation feedback loop which consists of the brain-and-muscle *arnt-*like protein 1 (*Bmal1*) gene and the circadian locomotor output cycle kaput (*clock*) gene [1]. Disruption of circadian clock function is closely associated with various diseases, including metabolic and gastrointestinal disorders [2], which are frequently accompanied by gut microbiota dysbiosis [3,4,5,6]. A bidirectional interaction exists between the host circadian clock and the gut microbiota. Circadian rhythm disturbances can significantly alter the diurnal oscillations of the gut microbiota [7]. Conversely, germ-free or antibiotic-treated mice exhibit disruptions in both peripheral and intestinal circadian rhythms, along with a loss of rhythmicity in metabolic outputs [8,9]. Supplementation with microbial metabolites, such as short-chain fatty acids (SCFAs), can in turn modulate the expression of core clock genes [10,11].

Disruption of the hepatic circadian clock is a key mechanism in the development of metabolic-associated fatty liver disease. Clock/Bmal1 heterodimers help maintain hepatic lipid homeostasis by regulating genes such as *PPARα* [12], while mutations in clock genes lead to severe hepatic steatosis in mouse models [13,14]. Guan et al. demonstrated through diet-induced remodeling of circadian enhancers how the circadian rhythm coordinates opposing hepatic lipid metabolic pathways (fatty acid synthesis and oxidation) [15]. At the same time, gut microbiota also regulates the process of lipid metabolism and it is also tightly related to diseases like obesity and fatty liver disease [16]. Host fat absorption and storage is modulated via modulation of the circadian function of intestinal epithelial cells [17].

Environmental factors, genetic factors, behavioral factors, etc., can all disrupt the balance of the circadian clock, lipid metabolism and gut microbiota. Nocturnal eating, eating at irregular times, and eating high-fat diets can disturb both the central and periphery body clock, change the gut bacteria, and also disturb the way our body processes fats [10,18,19]. Macronutrients of food like protein, fat, and carbs also seem to be able to adjust the circadian clock, gut bacteria, and metabolism all at once.

Recently, some studies have shown that some vitamin deficiencies, like those of A and D, could directly or indirectly disturb circadian rhythms and lipid metabolism. Vitamin A deficiency, for example, may result in the disruption of rhythmic output from the suprachiasmatic nucleus (SCN) and the disruption of locomotor rhythm, abnormal body temperature, and disrupted sleep–wake cycle [20]. Vitamin D deficiency can indirectly affect the circadian clock through gut microbiota homeostasis [21,22,23,24] while, at the same time, improving lipid metabolic disorder by regulating the gut–liver axis and inhibiting hepatocyte pyroptosis [25].

Vitamin E (VE) is a class of fat-soluble antioxidants and the most abundant form in the body is alpha-tocopherol (α-TE) [26]. Importantly, α-TE is the only form recognized by the α-TE transfer protein (TTP) in the liver, which selectively binds and retains α-TE, conferring it the highest biological activity among all vitamin E isoforms [26]. In addition to its antioxidative ability, α-TE has various physiological activities, such as modulating the immune system and offering anti-inflammatory and anti-atherosclerosis effects [27]. Studies showed that VE can reduce metabolic and inflammation abnormality occurring in NAFLD and NASH [28,29]. Clinical data show that VE intervention can significantly enhance the liver steatosis and inflammation of patients with NASH [30]. α-TE deficiency upregulates hepatic fatty acid transporter CD36, leading to intrahepatic fatty acid accumulation [31], whereas α-TE supplementation decreases hepatic CD36 expression and triglyceride (TG) content in animals on a high-fat diet [32]. α-TE deficiency can also cause health problems like lowered immunity and damaged neurological functions [27]. In recent years, some studies have begun to reveal the effect of α-TE on gut microbiota [33,34], though the systemic impact of its deficiency or high dose supplementation in healthy individuals is poorly understood.

To help close the knowledge gap, this study aimed to investigate the effects of short-term α-TE deficiency and high-dose α-TE supplementation on hepatic metabolism and gut microbiota in healthy adult mice, with a focus on the gut microbiota–liver axis. This study is expected to add new understanding regarding how α-TE may influence metabolic health, as well as offering possible theoretical grounding into nutritional approaches to synchronically affect biological clocks and gut microecology in the pursuit of maintaining metabolic homeostasis.

## 2. Materials and Methods

### 2.1. Design of Animal Experiments

Thirty-two 5-week-old male C57BL/6J mice were bought from Beijing Vital River Laboratory Animal Technology Co., Ltd (Beijing, China). All of the mice were placed in a room controlled at 24 ± 1 °C with a light and dark cycle of 12 h and were freely provided with food and water. After a 7-day adaptation period with a standard α-TE-normal purified diet, mice were placed in three groups based on their fasting body weight: a α-TE-deficient diet group (Deficiency, *n* = 8), a α-TE-normal diet group (Normal, *n* = 12), and a α-TE-supplementation diet group (Supplement, four times the normal α-TE level, *n* = 12). Mice with abnormal body weights were excluded to ensure no significant difference in the initial average body weight among groups (one-way ANOVA with LSD post hoc test, *p* < 0.05). Mice were housed two per cage. During the intervention period, fresh purified diets and sterilized deionized water were replenished every three days (between 09:00 and 10:00 a.m.), and food intake was recorded. Body weight was recorded every five days. On the morning (08:00) of day 36, mice from every group were euthanized by progressive CO_2_ asphyxiation in combination with cervical dislocation as a secondary confirmation method. Body weight, the net weight of each organ, and small intestine length were determined. Blood, liver, cecum, and cecal content were collected for the subsequent analysis. All of the procedures were approved by the life science ethics committee of Yunnan Agricultural University (No. 202207029, 22 July 2022).

### 2.2. Diet Formulation

In the animal experiments, all mice were fed purified diets formulated according to the AIN-93G standard. The Normal, Deficiency, and Supplement groups all had the same basal diet composition: 20% casein, 39.75% cornstarch, 13.2% maltodextrin, 10% sucrose, 5% cellulose, 7% walnut oil (used for oil-soluble additives), and 9% water (used for water-soluble additives). The experimental diets were produced by Jiangsu Mediscience Biomedical Co., Ltd (Yangzhou, China). (Table S1).

Walnut oil was collected from Yangbi County in Yunnan, China (Table S2). The walnut oil had 2.62 mg α-TE equivalents/100 g and was mostly composed of linoleic acid (64.35%) and oleic acid (18.02%). All of the nutrient analyses were carried out using the national food safety standard method of China [35,36] (GB 5009.168—2016 and GB 5009.82—2016).

The only difference among the three diets was the amount of dl-α-tocopheryl acetate added to the walnut oil (Table S3). The Normal group received 0.00782% (corresponding to a theoretical α-TE content of about 0.008% in the feed), the Deficiency group received 0% (theoretical α-TE content of approximately 0.0002%), and the Supplement group received 0.03183% (theoretical α-TE content of roughly 0.032% in the feed). All other nutritional components, including L-cystine (0.30%), compound mineral mix S10022G (3.50%), a VE-free vitamin mixture (1.00%), choline bitartrate (0.25%), and pigment (0.002%), remained consistent across groups.

### 2.3. Blood Biochemical Analysis

Fasting blood glucose levels in mice were measured before and after the dietary intervention using a portable glucometer (Bayer Contour, Mishawaka, IN, USA ). After the mice were sacrificed, the thoracic cavity was opened, and whole blood was taken from the abdominal aorta. The blood samples were centrifuged at 4000× *g* for 10 min at 4 °C to obtain the serum. The serum lipid analysis containing total cholesterol (TC), triglycerides (TGs), low-density lipoprotein cholesterol (LDL-C), and high-density lipoprotein (HDL-C) were detected by a commercially available biochemical testing kit purchased from Nanjing Jiancheng Bioengineering Institute. Serum and hepatic vitamin E concentrations were determined colorimetrically using the Vitamin E (VE) Colorimetric Assay Kit (Elabscience^®^, E-BC-K033-M, Wuhan, China).

### 2.4. Histopathological Examination

Livers were quickly harvested and put into 4 °C 10% neutral formalin. Following fixation, the tissue was dehydrated and paraffin embedded and sectioned at 3 μm. They were then stained with hematoxylin and eosin (H&E). Histological pictures were captured with an Olympus CX43 microscope with Cell Sens Entry (Olympus, Tokyo, Japan). The area ratio of ballooning degeneration was determined using Image-Pro Plus 6.0 image analysis software (Media Cybernetics Inc., Silver Spring, MD, USA). Measurements were performed on at least 10 independent microscopic fields per mouse liver section.

### 2.5. Liver RNA-Seq and Bioinformatics Analysis

Total RNA was extracted from mouse liver tissues, and its integrity and concentration were assessed using an Agilent 5300 Bioanalyzer (Agilent, Penang, Malaysia) and a NanoDrop-2000 (Thermo Fisher Scientific, Waltham, MA, USA), respectively. High-quality RNA samples (1 μg per sample) were used for library construction. Sequencing libraries were prepared using the Illumina Stranded mRNA Prep, Ligation Kit (Illumina, San Diego, CA, USA) following the manufacturer’s protocol. Library quantification was performed on a Qubit 4.0 Fluorometer (Thermo Fisher Scientific: Waltham, MA, USA), and paired-end sequencing (2 × 150 bp) was conducted on an Illumina NovaSeq X Plus platform. All library preparation and sequencing services were provided by Shanghai Majorbio Bio-pharm Biotechnology Co., Ltd (Shanghai, China).

Raw sequencing reads were subjected to quality control, including the removal of low-quality bases and adapter sequences. Clean reads were then aligned to the mouse reference genome (e.g., GRCm39) using an appropriate aligner (e.g., HISAT2). Gene expression levels were quantified, and differentially expressed genes (DEGs) between comparison groups were identified using standard bioinformatics tools (DESeq2, with thresholds such as |log_2_FC| > 1 and *p*_adjust_ < 0.05). Functional annotation and enrichment analysis of DEGs were performed based on the KEGG database.

### 2.6. RNA Extraction and Quantitative Real-Time PCR (qPCR)

Total RNA was extracted from mouse liver tissue using the TaKaRa MiniBEST Universal RNA Extraction Kit (9767, TaKaRa, Dalian, China). RNA concentration and purity were assessed with a TGem Pro ultramicro UV-Vis spectrophotometer (Tiangen, Beijing, China). Complementary DNA (cDNA) was synthesized from 1 μg of total RNA using the PrimeScript™ RT Reagent Kit with gDNA Eraser (TaKaRa, RR047A, Dalian, China), following the manufacturer’s instructions. Quantitative PCR was performed on a LightCycler 480 System (Roche Diagnostics) with TB Green™ Premix ExTaq™II (TaKaRa, RR820A, Dalian, China). Gene expression levels were normalized and calculated using the 2^−ΔΔCt^ method. Primer sequences are listed in Table S4.

### 2.7. Western Blot Analysis

Mouse liver tissues were homogenized and lysed on ice using RIPA lysis buffer (Solarbio, R0010, Beijing, China) supplemented with 1 mM PMSF and phosphatase inhibitors. The lysates were centrifuged, and the supernatant protein concentration was quantified using a NanoDrop-like protein quantitation system (Life Real, FC-3100, Hangzhou, China). An aliquot containing 120 μg of total protein was mixed with 5× loading buffer, denatured at 95 °C, and subjected to SDS-PAGE. Gels were prepared using the Omni-Easy™ One-Step PAGE Gel Fast Preparation Kit (EpiZyme, PG212, Shanghai, China) and electrophoresis was performed using a Bio-RAD system at 150 V for 50 min. Proteins were then transferred onto methanol-activated PVDF membranes (Millipore, IPVH00010, 0.45 μm, Darmstadt, Germany). Following transfer, the membranes were blocked with 5% skim milk in TBST at room temperature for 1.5 h.

The membranes were incubated overnight at 4 °C with primary antibodies diluted in blocking buffer. After washing with TBST, membranes were incubated with corresponding HRP-conjugated secondary antibodies (Proteintech, Wuhan, China) at room temperature for 1.5 h. Primary antibodies used were as follows: rabbit anti-Arntl monoclonal antibody (Proteintech, 86113-1-RR, 1:5000), rabbit anti-CLOCK monoclonal antibody (Proteintech, 82829-1-RR, 1:5000), mouse anti-PER2 monoclonal antibody (Proteintech, 67513-1-Ig, 1:5000), rabbit anti-Scd1 polyclonal antibody (Proteintech, 28678-1-AP, 1:4000), and rabbit anti-GAPDH polyclonal antibody (Proteintech, 10494-1-AP, 1:5000). Protein bands were visualized using Millipore chemiluminescent substrate (WBKLS0100, Merck Millipore, Darmstadt, Germany) and imaged with a Bio-Rad Chemidoc X+ imaging system. Protein levels were normalized to *β-actin* as a loading control.

### 2.8. 16S rRNA Gene Sequencing and Gut Microbiota Bioinformatics Analysis

Cecal content metagenomic DNA was extracted using the E.Z.N.A.^®^ soil DNA kit (Omega Biotek, Norcross, GA, USA). The V3–V4 hypervariable regions of the bacterial 16S rRNA gene were amplified via PCR with primers 338F and 806R. The amplicons were purified, quantified, and paired-end sequenced on an Illumina platform by Shanghai Majorbio Biomedical Technology Co., Ltd (Shanghai, China). Raw sequencing reads were deposited in the NCBI SRA database.

Bioinformatic processing was performed using QIIME 2 (v2022.2). Sequences were quality-filtered, denoised with DADA2, and clustered into amplicon sequence variants (ASVs). Taxonomic assignment of ASVs was conducted using a naive Bayes classifier against the SILVA 16S rRNA database (v138.2). To ensure comparability, all samples were rarefied to an equal sequencing depth per sample. Alpha diversity indices were compared across groups using the one-way ANOVA followed by post-hoc tests (Dunn’s *t* test). Beta diversity, based on Bray–Curtis distances, was visualized via principal coordinate analysis (PCoA). Differential taxa among groups were identified with linear discriminant analysis effect size (LEfSe) (LDA score > 2.0, *p* < 0.05).

### 2.9. Statistical Analysis

All experimental data were expressed as the mean ± standard error of the mean (SEM). Statistical analysis was done with GraphPad Prism (version 8.0) and SPSS (version 22.0) software. One-way ANOVA was used to test for difference between groups. When there was a significant effect, post hoc multiple comparisons were applied. For the data which did not satisfy the assumption of the parametric test, the non-parametric Kruskal–Wallis H test was adopted. The significance levels were set at *p* < 0.05. Multivariate analyses of the gut microbiota, including principal coordinate analysis (PCoA) and linear discriminant analysis effect size (LEfSe), were performed on the Majorbio Cloud Platform (http://www.majorbio.com/). Correlations between gut bacterial genera and host parameters were evaluated using Spearman’s rank correlation coefficient in SPSS. Heatmaps were generated using HemI (version 1.0) and Adobe Illustrator 2022.

## 3. Results

### 3.1. Short-Term α-TE Deficiency Adversely Affects Liver and Intestinal Indices in Mice

**Figure 1:**
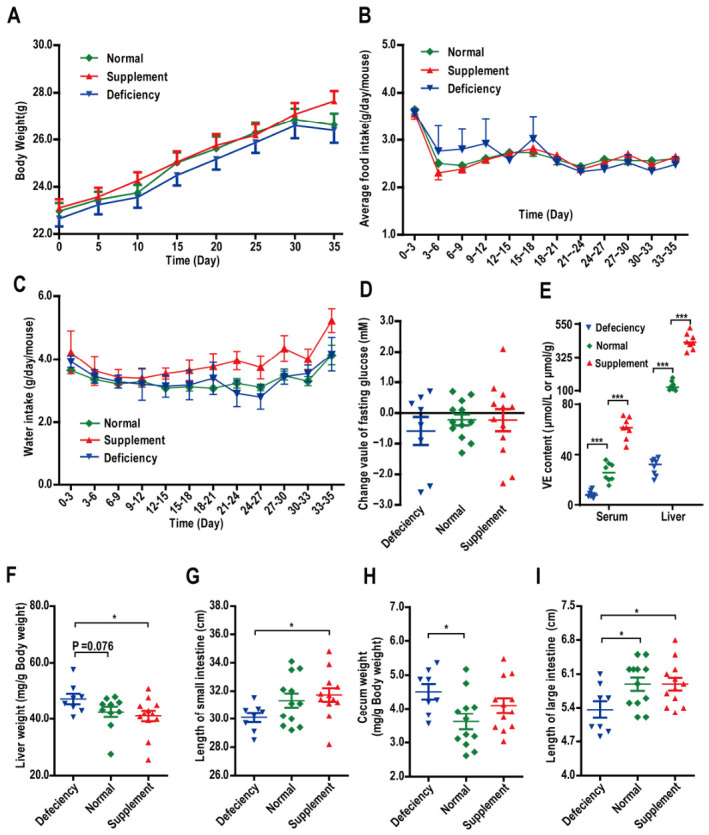
Effects of α-TE on body weight, dietary intake, blood glucose, and organ indices in mice. (**A**) Body weight changes, (**B**,**C**) average daily food and water intake, (**D**) fasting blood glucose levels, (**E**) α-TE levels in both serum and liver tissue, (**F**) liver index, (**G**) small intestine length, (**H**) cecum weight, (**I**) large intestine length. Data are presented as mean ± SEMs; *n* = 8 or 12. Statistical significance between groups was determined by one-way ANOVA followed by post hoc analysis. * indicates a significant difference between the two groups. *, *p* < 0.05; ***, *p* < 0.001.

dl-α-tocopheryl acetate is the most common form added to animal feed as a substitute for α-TE. Once ingested, it is hydrolyzed to produce α-TE. In animal experiments, although five consecutive weeks (35 days) of α-TE treatment did not significantly affect mouse body weight, fasting blood glucose, or food intake (Figure 1A–D), it significantly altered α-TE levels in both blood and liver tissue (Figure 1E). Serum and hepatic α-TE concentrations were significantly lower in the α-TE-deficient group when compared with the Normal group, while the Supplement group showed elevated levels, confirming the efficacy of the dietary intervention. Furthermore, α-TE treatment significantly influenced liver and intestinal indices (Figure 1F–I). Mice in the α-TE-deficient group showed a significant increase in liver (Figure 1F) and cecal indices (Figure 1H), as well as a significant shortening of the small intestine (Figure 1G) and colorectal length (Figure 1I).

**Figure 2:**
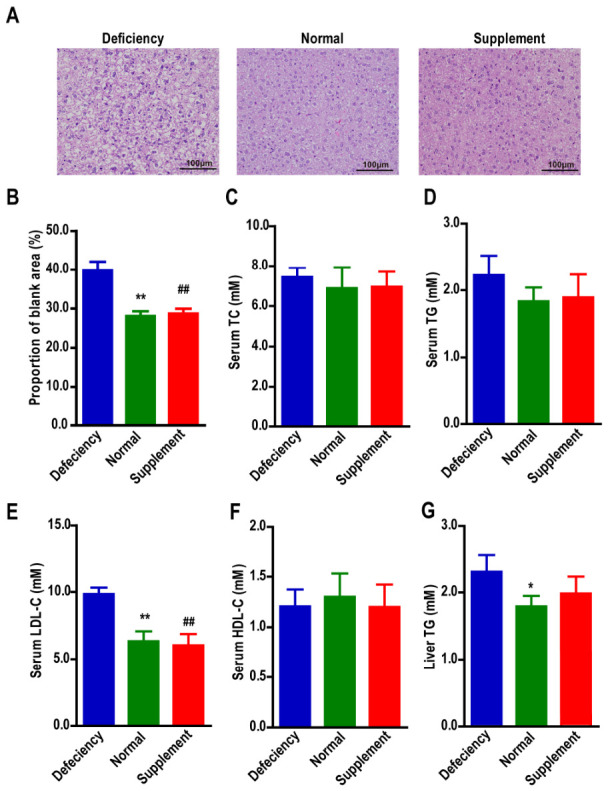
Effects of α-TE-deficient diet on liver histomorphology and serum lipid profile in mice. (**A**) Representative liver histology (H&E staining); (**B**) percentage area of blank regions in H&E-stained liver sections; (**C**–**F**) serum levels of total cholesterol (TC, (**C**)), triglycerides (TG, (**D**)), low-density lipoprotein cholesterol (LDL-C, (**E**)), and high-density lipoprotein cholesterol (HDL-C, (**F**)); (**G**) Liver TG. Data are expressed as mean ± SEMs; *n* = 8. Statistical significance was assessed by one-way ANOVA followed by post-hoc tests. * indicates a significant difference between the Normal and Deficiency groups; # indicates a significant difference between the Supplement and Deficiency groups. *, *p* < 0.05; **, *p* < 0.01; ##, *p* < 0.01.

The liver is the most important metabolic organ in the body. HE staining results indicated that mice in the α-TE-deficient group exhibited a certain degree of “ballooning degeneration” in the liver (Figure 2A), with a significant increase in the proportion of blank areas in tissue sections (Figure 2B). The results of the lipid profile tests (Figure 2C–F) showed that α-TE deficiency led to a significant increase in serum LDL-C (Figure 2E) and liver TG levels (Figure 2G). In contrast, α-TE supplementation had no significant effect on the lipid parameters (Figure 2C–F). Collectively, these data imply that a systemic impact on overall liver metabolism may have already occurred within five weeks of α-TE deficiency.

### 3.2. α-TE-Deficient Diet Affects the Liver Transcriptome in Mice

**Figure 3:**
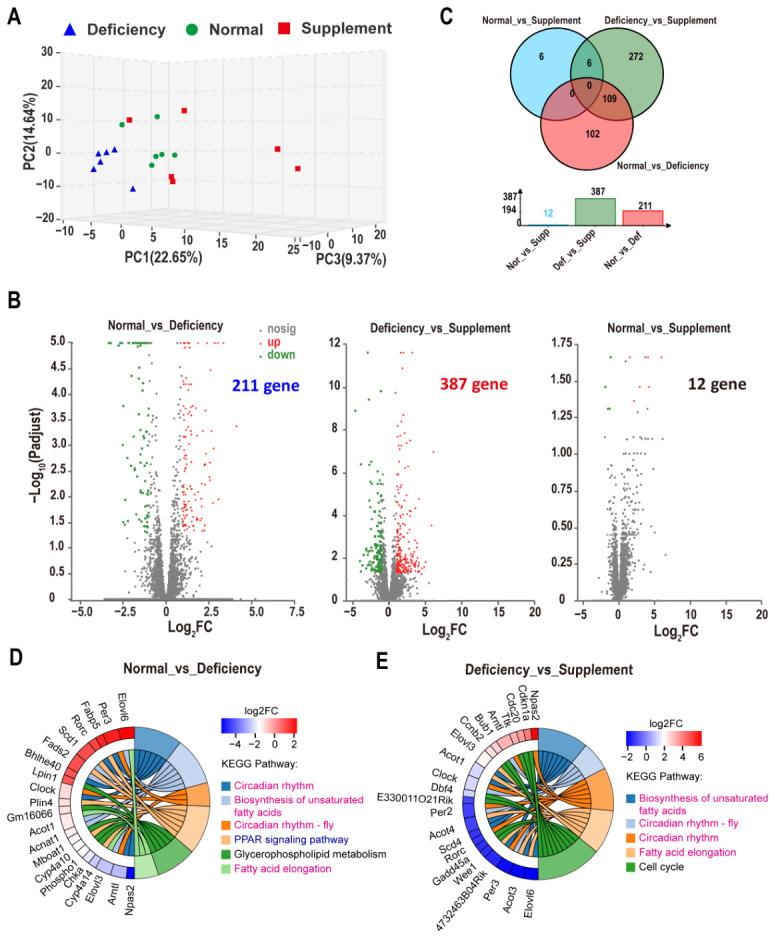
Effect of α-TE-deficient diet on the mouse liver transcriptome. (**A**) Principal component analysis (PCA) of liver RNA-seq gene expression profiles (*n* = 8). (**B**) Volcano plot of differentially expressed genes. Red dots represent significantly upregulated genes, green dots represent significantly downregulated genes (DESeq2, *p*_adjust_ < 0.05). (**C**) Venn diagram showing shared and unique differentially expressed genes between comparison groups. (**D**,**E**) Chord plots of KEGG enrichment analysis results.

Given the significant impact of the α-TE-deficient diet on liver indices and tissue morphology in mice, we examined the liver transcriptome using RNA-Seq technology. Principal component analysis of the liver transcriptome sequencing results revealed that the α-TE-deficient diet shaped a markedly distinct gene expression pattern (Figure 3A). A total of 211 significantly differentially expressed genes were identified between the Deficiency and Normal groups, 387 between the Deficiency and Supplement groups, and only 12 between the Normal and Supplement groups (Figure 3B). Further analysis showed a high proportion of overlapping significantly differentially expressed genes between the Deficiency and Normal groups and the Deficiency and Supplement groups (109 genes, Figure 3C). These data indicate that α-TE deficiency significantly altered the liver transcriptome.

### 3.3. α-TE-Deficient Diet Disrupts Hepatic Circadian Clock and Lipid Metabolism

KEGG enrichment analysis of the differentially expressed gene sets between the two groups revealed that the differential genes between the Deficiency and Normal groups were specifically enriched in signaling pathways such as circadian rhythm (circadian rhythm-fly, circadian rhythm), peroxisome proliferator-activated receptor signaling pathway (PPAR signaling pathway), fatty acid elongation, biosynthesis of unsaturated fatty acids, and glycerophospholipid metabolism (Figure 3D). Similarly, the significantly differentially expressed genes between the Deficiency and Supplement groups were also enriched in pathways related to circadian clock, fatty acid elongation, and biosynthesis of unsaturated fatty acids (Figure 3E).

**Figure 4:**
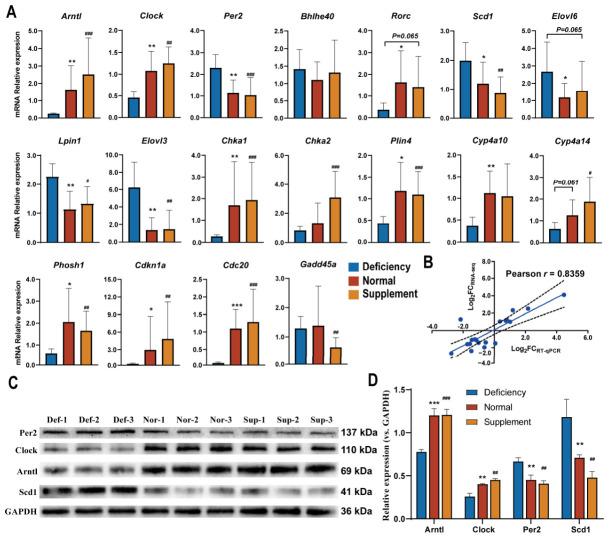
α-TE-deficient diet induces hepatic circadian clock and lipid metabolism disorders in mice. (**A**) RT-qPCR validation of selected differentially expressed genes identified from KEGG enrichment analysis (*n* = 6); (**B**) correlation between RNA-Seq and RT-qPCR results; (**C**,**D**) protein expression levels of Arntl (Bmal1), Clock, Per2, and Scd1 in liver tissue (*n* = 3). Data are presented as mean ± SD. Statistical significance was determined by one-way ANOVA followed by post-hoc tests. * indicates a significant difference between the Normal and Deficiency groups; # indicates a significant difference between the Supplement and Deficiency groups. *, *p* < 0.05, **, *p* < 0.01, ***, *p* < 0.001; #, *p* < 0.05, ##, *p* < 0.01, ###, *p* < 0.001.

Therefore, we used the RT-qPCR method to examine the expression of core genes involved in hepatic circadian clock, as well as lipid synthesis, modification, storage, breakdown, and oxidation pathways. The results were found to be largely consistent with the differential gene expression obtained from RNA-Seq analysis (Figure 4A), and the two detection methods showed a significant correlation (*r* = 0.8359, Figure 4B), indicating that the α-TE-deficient diet significantly affected the expression of genes related to hepatic circadian clock and lipid metabolism.

To further confirm the disruption of hepatic circadian clock and lipid metabolism caused by the α-TE-deficient diet, we performed Western blot analysis to assess the expression of core circadian clock proteins—Arntl (Bmal1), Clock, and Per2—along with the key lipogenic enzyme Scd1 in mouse livers. The results showed that the α-TE-deficient diet significantly suppressed the expression of the “positive regulatory arm” proteins Arntl and Clock, upregulated the expression of the negative regulatory arm protein Per2, and also increased the expression of Scd1 (Figure 4C,D). These findings suggest that the α-TE-deficient diet may induce abnormalities in liver indices and tissue morphology in mice by disrupting hepatic circadian clock and lipid metabolism.

### 3.4. α-TE Deficiency Reduces Cecal Microbial Diversity and Alters Its Composition

**Figure 5:**
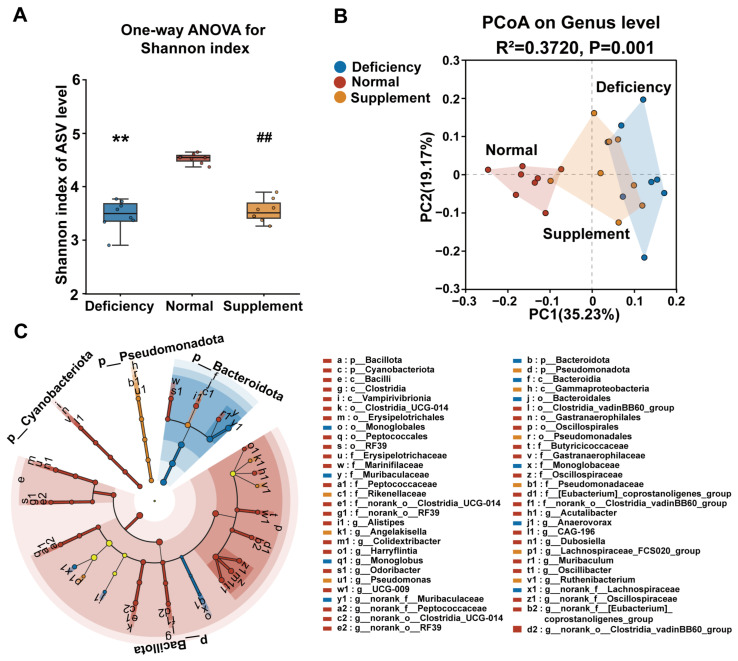
α-TE alters the structure and composition of the microbial community in the mouse cecum. (**A**) α-diversity index (Shannon); (**B**) β-diversity analysis via principal coordinate analysis (PCoA, based on Bray–Curtis distance); (**C**) multi-level species cladogram derived from LEfSe (LDA score > 2.0) analysis, displaying taxonomic hierarchy from phylum to genus. Data are presented as mean ± SD. Statistical significance was determined by one-way ANOVA followed by post-hoc tests (Dunn’s *t* test). * indicates a significant difference between the Normal and Deficiency groups; # indicates a significant difference between the Supplement and Normal groups. **, *p* < 0.01; ##, *p* < 0.01.

Given that α-TE deficiency significantly affects intestinal indices, including a notable increase in the cecal index (Figure 1H) and a significant shortening of the small intestine (Figure 1G) and colorectal length (Figure 1I), we used 16S rRNA gene high-throughput sequencing technology to assess the microbial diversity in the mouse cecum. The results showed that the α-TE deficient diet greatly decreased the alpha diversity of the cecal microbiota, Figure S1, as indicated by a notable decrease in the Shannon index (Figure 5A). PCoA analysis showed that the α-TE -deficient diet also changed the beta diversity of cecal microbiota in mice (Figure 5B). It is worth mentioning that excessive α-TE supplementation had a similarly compromised impact on the microbial diversity in the mouse cecum (Figure 5A,B).

**Figure 6:**
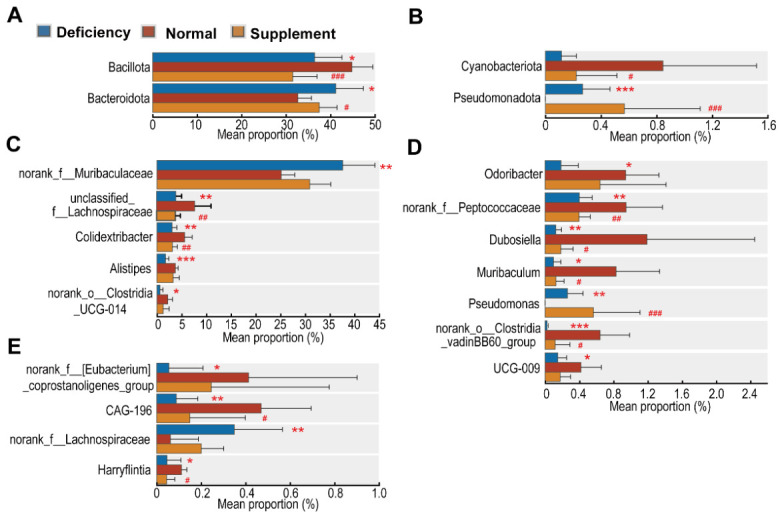
Cecal microbial taxa in mice significantly influenced by α-TE-deficient and supplemented diets (phylum and genus levels). (**A**,**B**) Differentially abundant taxa at the phylum level; (**C**–**E**) differentially abundant taxa at the genus level. Data are expressed as mean ± SD. Statistical significance was determined using the Kruskal–Wallis H test followed by Dunn’s post hoc test. * *p* < 0.05, ** *p* < 0.01, *** *p* < 0.001 for the Deficiency group vs. the Normal group; # *p* < 0.05, ## *p* < 0.01, ###*p* < 0.001 for the Supplement group vs. the Normal group.

In terms of species composition, multi-level species discriminant analysis (LEfSe) revealed that many microbial taxa were significantly influenced by α-TE from the phylum to genus levels (Figure 5C), including 4 phyla, 5 classes, 10 orders, 13 families, and 24 genera (Figure 5C). At the phylum level, the α-TE-deficient diet significantly reduced the relative abundance of the dominant taxon Bacillota, while increasing the relative abundance of the dominant taxa Bacteroidota and Pseudomonadota (Figure 6A,B). Excessive α-TE supplementation also exerted similar effects on these three phyla in the mouse cecum.

At the genus level, several dominant taxa were also significantly affected by α-TE treatment (Figure 6C). The α-TE-deficient diet significantly increased the relative abundance of the highly abundant taxon *norank_f__Muribaculaceae* (*p* < 0.01), as well as that of the low-abundance taxa *Pseudomonas* and *norank_f__Lachnospiraceae* (Figure 6D,E). In stark contrast, numerous microbial taxa, including both high- and low-abundance groups, were significantly reduced under the influence of the α-TE-deficient diet. Examples include the *unclassified_f__Lachnospiraceae*, *Colidextribacter*, *Alistipes*, and *norank_o__Clostridia_UCG-014* shown in Figure 6C; *Odoribacter*, *norank_f__Peptococcaceae*, *Dubosiella*, *Muribaculum*, *norank_o__Clostridia_vadinBB60_group*, and *UCG-009* shown in Figure 6D; and *norank_f__[Eubacterium]_coprostanoligenes_group*, *CAG-196*, *norank_f__Lachnospiraceae*, and *Harryflintia* shown in Figure 6E.

### 3.5. Perturbation of the Hepatic Circadian Clock and Lipid Metabolism Significantly Correlates with Changes in the Gut Microbiota

Based on the significant perturbation of the hepatic circadian clock, hepatic lipid metabolism, and gut microbiota induced by α-TE-deficient diet, bivariate correlation analysis were used to establish the correlations between the specific gut bacterial genera and core genes.

**Figure 7:**
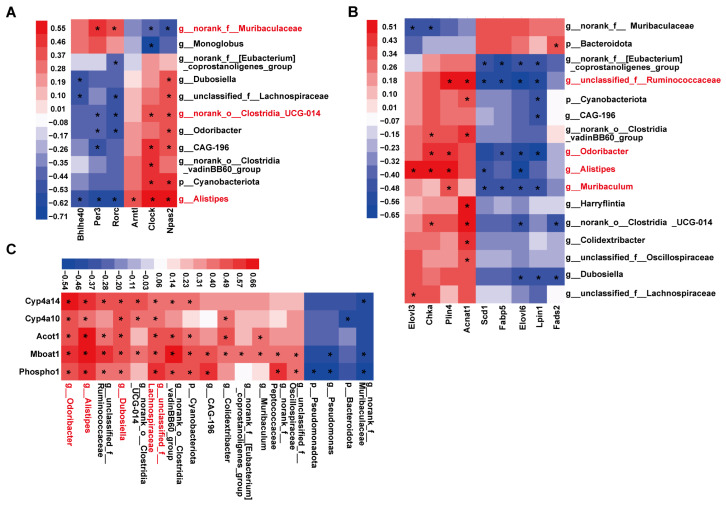
Heatmaps depicting correlations between specific gut bacterial genera and key hepatic circadian clock and lipid metabolism genes. (**A**) Gut bacteria versus core hepatic circadian clock genes. (**B**) Gut bacteria versus core genes involved in lipid synthesis, modification, and storage. (**C**) Gut bacteria vs. core genes for lipid catabolism and oxidation. Correlation was done using Spearman’s rank correlation (*n* = 6 per group). Color intensity corresponds to the *r* value. The *p* values have been adjusted with the *Bonferroni–Hochberg* method. * indicates significance (*p* < 0.05).

As shown in Figure 7A, multiple genera exhibited significant correlations with key hepatic circadian clock genes, including *Arntl*, *Bhlhe40*, *Clock*, *Per3*, *Npas2*, and *Rorc*. Notably, *Alistipes*, *norank_f__Muribaculaceae*, and *norank_o__Clostridia_UCG-014* associated significantly with over four core hepatic circadian clock genes. *Alistipes* showed important correlations with all six different hepatic circadian clock genes mentioned above (*p* < 0.05). *Alistipes* was positively correlated with *Arntl*, *Clock*, and *Npas2*, and negatively correlated with *Bhlhe40*, *Per3*, and *Rorc.* These findings indicate that genera like *Alistipes* may be important for hepatic circadian clock disruption due to an α-TE-deficient diet.

Core genes participating in lipid synthesis, alteration, and storage were also correlated to bacterial genera through the use of Spearman’s method. As seen in Figure 7B, several genera were associated with these core genes. *Alistipes*, *unclassified_f__Ruminococcaceae*, *Odoribacter,* and *Muribaculum* are significantly correlated with more than five core genes related to lipid synthesis, modification and storage. It is worth noting that *Alistipes* is greatly negatively correlated with *Scd1* and *Elovl6* but positively correlated with *Elovl3*, *Chka* and *Plin4*. In the same way, *Muribaculum* has very significant negative connections with *Scd1*, *Fabp5*, *Elovl6*, and Lpin1, but a positive one with *Plin4*. These findings imply that genera including *Alistipes* and *Muribaculum* may play a role in the dysregulation of hepatic lipid synthesis, modification, and storage triggered by α-TE.

The transcriptome analysis results tell us that the α-TE-deficient diet also affects hepatic lipid catabolism and oxidation. Hence, we explored the correlations among differentially abundant bacterial genera and hepatic lipid catabolism-related genes and hepatic lipid oxidation-related genes. There were many differentially expressed bacterial genera, which were correlated with the *Cyp4a14*, *Cyp4a10*, *Acot1*, *Mboat1*, and *Phospho1* genes (Figure 7C). Among these, *Alistipes*, *Odoribacter*, *Dubosiella*, and *unclassified_f__Lachnospiraceae* were especially noteworthy, as they had very strong positive correlations with all five aforementioned genes. These findings indicate that the genera of *Alistipes*, *Odoribacter*, *Dubosiella*, and *unclassified_f__Lachnospiraceae* might be associated with the hepatic lipid catabolic process and the hepatic oxidative process, respectively, which are caused by α-TE deficiency.

## 4. Discussion

The present study demonstrates that short-term α-TE deficiency is sufficient to induce hepatic steatosis, disrupt circadian clock gene expression, and perturb lipid metabolism in healthy adult mice, and that these alterations are closely linked to a marked restructuring of the cecal microbiota. Specifically, five weeks of α-TE deficiency led to adverse phenotypic changes, including increased liver and cecal indices, shortened intestinal length, hepatic ballooning degeneration, and elevated serum LDL-C and hepatic TG levels. Mechanistically, α-TE deficiency suppressed the positive-arm clock genes *Arntl* (*Bmal1*) and *Clock*, while upregulating the negative-arm regulator *Per2*. This circadian disruption was accompanied by coordinated activation of de novo lipogenesis and lipid modification pathways (e.g., upregulation of *Scd1*, *Elovl6/3*, *Lpin1*) together with the inhibition of fatty acid oxidation pathways (e.g., downregulation of *Cyp4a10/14*, *Acot1*), a metabolic shift that favors intrahepatic lipid accumulation. In support of the gut–liver axis mechanism, 16S rRNA sequencing revealed that α-TE deficiency significantly reduced cecal microbial α-diversity and altered species composition. Spearman correlation analysis further identified several bacterial genera, particularly *Alistipes*, as significantly correlated with the expression of hepatic circadian and lipid metabolic core genes. Collectively, these findings suggest that α-TE deficiency may reshape gut microbiota composition, thereby disrupting the ‘gut microbiota–liver circadian clock’ signaling dialogue and reprogramming hepatic lipid metabolism. This study thus provides a multi-omics perspective linking short-term vitamin E restriction to metabolic disturbances via the gut–liver axis.

The center of the circadian clock is a self-oscillatory system depending on a transcription–translation negative feedback loop, mainly existing in the SCN and peripheral organs like the liver. This loop starts up when Arntl/Clock heterodimer starts the transcription of genes like *Per* and *Cry*. Subsequently, the translated Per/Cry proteins feed back to inhibit their own transcription. Following protein degradation, this triggers another round of transcription, and thus establishes an oscillation with a period [37,38]. Moreover, auxiliary regulatory components like Npas2, Bhlhe40, Rorc, and others fine tune, stabilize, and integrate external signals within the core loop so that loop operates precisely [39,40,41]. Of these, Arntl/Clock heterodimers serves as the main transcriptional engine, and its downregulation breaks the entire downstream transcriptional program. Our data demonstrate that α-TE deficiency suppresses Arntl/Clock while upregulating Per2 and also alters the mRNA expression of regulators including *Npas2*, *Bhlhe40*, and *Rorc*. These results collectively establish that α-TE deficiency directly disrupts the core molecular machinery of the circadian clock.

Circadian disruption is usually connected with metabolic disorders. Previous studies have found that when liver-specific *Bmal1* is knocked out, intrahepatic TG accumulates [42,43]. Similar to the previous study, deficiency of α-TE in the present study also resulted in a clear increase in the content of TG in the liver and ballooning degeneration in the histology. Gene expression analysis showed that the α-TE deficiency-induced lipid synthesis pathway went all the way from creation to storage: *Scd1* (desaturation) and *Elovl6/3* (elongation) were for fatty acid de novo production and modification [44,45,46], while *Lpin1* carried out diacylglycerol production, an important part of TG synthesis [47]. Diacylglycerol can be further taken up by enzymes like *Chka* and directed to make either phospholipid or TG [48]; finally, TG is packed up into lipid droplets by *Plin4* [49] for storage. The coordinated upregulation of these genes (*Scd1*, *Elovl6*, and *Lpin1*) suggests that the liver is in a pronounced net lipogenic and storage state.

Simultaneously, α-TE deficiency broadly turned down the key genes concerning lipid catabolism and oxidation pathways. The downregulation of *Cyp4a10/14* could impair the liver’s ability to clear extra fatty acids [50]. The reduction in *Acot1* expression might limit fatty acid intake by mitochondria for β-oxidation [51,52]. In addition, the decreased amounts of *Phpspho1* and *Mboat1* may interfere with the membrane conditions required for oxidative reactions [53,54]. These genes all belong to a connected catabolic network, and their simultaneous inhibition will directly cause the accumulation of fatty acid inside the hepatocyte. More importantly, α-TE deficiency also remarkably decreased the expression of the nuclear receptor gene *Rorc. Rorc* can synergize with *Bmal1/Clock* to activate many catabolic genes and potentially inhibit certain synthetic genes [12,55]. Consequently, the downregulation of *Rorc* may contribute to a worsening of metabolic imbalance. Collectively, α-TE deficiency contributes to intrahepatic TG accumulation via the concurrent upregulation of lipogenic pathways and downregulation of fatty acid oxidative pathways.

The gut microbiota has its own circadian rhythmicity, which is regulated by the host circadian clock. In turn, microbiota-derived metabolites, such as SCFAs and bile acids also give feedback for the host clocks. This bidirectional interaction forms a “microbiota-metabolite-clock” regulatory axis that is crucial for liver metabolism [56]. There is a bidirectional relationship between α-TE and gut microbiota. Low-dose α-TE feeding can change the structure of gut microbiota and body weight [34], and microbiota also participate in the metabolism of α-TE, affecting its bioavailability [57]. In this work we can see that both α-TE deficiency and high dose supplementation significantly regulated the cecal microbiota structure, and these changes were strongly related to the hepatic circadian clock and lipid metabolism. Additionally, the shift of some key bacterial genera are worth mentioning: *Alistipes*, *norank_f__Muribaculaceae*, *Muribaculum*, *Odoribacter*, and *Dubosiella*.

*Alistipes* is a common genus in healthy adult guts and can produce metabolites like butyrate. Human metagenomic studies have indicated that *Alistipes* was found more often in individuals with regular, “lark”-type chronotypes, suggesting an association with healthy circadian rhythms [58]. Regarding lipid metabolism, some strains of *Alistipes* possess unique lipid-processing capabilities [59] and can reduce the hepatic lipid accumulation by improving intestinal barrier function [60]. Thus, it might be a major hub linking α-TE deficiency, circadian disruption, and lipid metabolic abnormalities.

The relative abundance of *norank_f__Muribaculaceae* was enriched in α-TE deficiency and correlated with clock genes, *Muribaculum* decreased in abundance and was associated with lipogenic genes. Host *Bmal1* knockout could lead to a decreased abundance of Muribaculaceae [61], indicating the shaping role of the host clock on the microbiota. They might be intermediating for the disturbance that α-TE deficiency caused.

*Odoribacter* acts like a “lipid chemist” and takes part in metabolism and manipulation of bile acids and manipulation of lipid milieu [62,63,64]. Additionally, it has been proved that *Dubosiella* can also improve hepatic lipid metabolism through the “FGF21–dubosilla” axis [65,66]. The α-TE deficiency greatly reduced the abundance of both genera, and this is consistent with the known metabolic protective role that both have; thus, they may play a role in the disturbances that arise from α-TE deficiency.

In summary, this study reveals a potential novel mechanism whereby α-TE deficiency may disrupt the homeostasis of the hepatic core circadian clock by altering the composition of gut microbiota, including key genera such as *Alistipes*, thereby disrupting the gut-to-liver circadian signaling dialogue. This disruption leads to a reprogramming of hepatic lipid metabolism, characterized by a dual imbalance of enhanced lipid synthesis and attenuated oxidation, which finally drives the intrahepatic lipid accumulation. This study adopted a multi-omics integration strategy to construct a correlation network between gut microbiota and host hepatic metabolism. In addition, a purified diet model was used, allowing precise manipulation of α-TE while controlling for confounding variables. Nevertheless, this study has certain limitations. For example, our data only reflect the effect of α-TE on the expression of circadian clock-related genes at a single time point, rather than a systematic effect on circadian amplitude, phase, or period, which warrants further investigation. Moreover, future in-depth studies should focus on causal validation, metabolomic analysis, and the long-term effects of α-TE deficiency. Addressing these limitations will further strengthen the causal link between α-TE status, gut microbiota, and hepatic circadian metabolic regulation.

## 5. Conclusions

It was proved in this study that a five-week α-TE deficiency caused significant adverse effects on the liver and intestines of healthy adult mice. These effects resulted in elevated liver and cecum indices, shortened length of small intestine and colorectum, and abnormal hepatic lipids. Transcriptomic, RT-qPCR, and Western blot results further demonstrated that α-TE deficiency disturbed hepatic circadian clock, upregulated hepatic lipid synthesis, but suppressed fatty acid oxidation and catabolic pathways. Results of the 16S rRNA gene sequencing showed that α-TE deficiency greatly decreased cecal microbiota diversity and greatly changed the species composition of the cecum. Correlation analysis implied that bacterial genera, including *Alistipes* and *norank_f__Muribaculaceae*, may play an important role in the disruption of the hepatic circadian clock and lipid metabolism in α-TE deficiency. The absence of α-TE has disrupted the balance of gut microbiota, and in turn affected the function of the main liver circadian clock due to changes in the make-up of gut microbiota. This circadian disarray directly resulted in a reprogramming of hepatic lipids and, eventually, intrahepatic lipids and early-stage injury. These findings reveal the critical importance of α-TE intake in maintaining metabolic homeostasis through the “microbiota-clock” axis and provide a theoretical foundation for the nutritional prevention of metabolic diseases.

## Data Availability

The raw reads of 16S rRNA gene and RNA-seq sequence data were deposited into the NCBI Sequence Read Archive (SRA) database under BioProject accession numbers PRJNA1390973 and PRJNA1392694 respectively.

## Supplementary Materials

The following supporting information can be downloaded at https://www.mdpi.com/article/10.3390/nu18121853/s1, Tables S1: Main nutritional components of mouse feed; Tables S2: Main fatty acids and Vitamin E content in walnut oil; Tables S3: Main nutritional components added to feed via water and oil; Tables S4: Primers sequences used for quantitative PCR analysis of gene expression.

## Abbreviations

VE	Vitamin E
Bmal1/Arntl	Aryl hydrocarbon receptor nuclear translocator-like
Clock	Clock circadian regulator
Per2	Period circadian regulator 2
Rorc/Rorγ	Nuclear receptor subfamily 2 group F member
Npas2	Neuronal PAS domain protein 2
Bhlhe40/Dec1	Basic helix-loop-helix family member e40
Fabp5	Fatty acid-binding protein 5
PPAR	Peroxisome proliferator-activated receptor
Scd1	Stearoyl-CoA desaturase-1
Elovl3/6	Fatty acid elongase 3/6
Chka	Choline kinase alpha
Plin4	Perilipin 4
Cyp4a10	Cytochrome P450 family 4 subfamily A member 10
Cyp4a14	Cytochrome P450 Family 4 Subfamily A member 14
Acot1	Acyl-CoA thioesterase 1
Mboat1	Membrane-bound O-acyltransferase domain containing 1
Phospho1	Phosphoethanolamine/phosphocholine phosphatase 1
LDL-C	Low-density lipoprotein cholesterol
TG	Triglyceride
PCA	Principal component analysis
PCoA	Principal coordinates analysis
LEfSe	Linear discriminant analysis effect size
SCFAs	Short-chain fatty acids
