# Supplementary material for: Dietary α-Tocopherol Deficiency Disrupts Hepatic Circadian Clock and Lipid Metabolism in Association with Gut Microbiota Dysbiosis
Source: Nutrients. 2026 Jun 9;18(12):1853. doi: 10.3390/nu18121853 (PMC13305107; doi:10.3390/nu18121853)
Supplement: Supplementary file 1 [file nutrients-18-01853-s001.zip › nutrients-4322516-supplementary.pdf]

Dietary α-Tocopherol Deficiency Disrupts Hepatic Circadian Clock and Lipid Metabolism in Association with Gut Microbiota Dysbiosis

Lei Peng[a,b,c*], Yan Zhao[d*], Yuqin Fan[a,b,c], Qi Peng[a,b,c], Jun Sheng[c], Yang Tian[a,b,c#], Xiaoyu Gao[a,b,c#]

a Yunnan Key Laboratory of Precision Nutrition and Personalized Food Manufacturing, Yunnan Agricultural University, Kunming 650201, China

b Engineering Research Center of Development and Utilization of Food and Drug Homologous Resources, Ministry of Education, Yunnan Agricultural University, Kunming 650201, China

c College of Food Science and Technology, Yunnan Agricultural University, Kunming 650201, China

d Division of Science and Technology, Yunnan Agricultural University, Kunming 650201, China

* contributed equally to this work.

# Address correspondence to Xiaoyu Gao, 2018014@ynau.edu.cn; Yang Tian, tianyang@ynau.edu.cn.

Supplementary tables and figures

Table S1 Main nutritional components of mouse feed

Ingredient	Normal	Deficiency	Supplement
Casein (80 mesh)	20.00%	20.00%	20.00%
Corn starch	39.75%	39.75%	39.75%
Maltodextrin (DE=10)	13.20%	13.20%	13.20%
Sucrose	10.00%	10.00%	10.00%
Cellulose	5.00%	5.00%	5.00%
Walnut oil (containing oil-soluble additives)	7.00%	7.00%	7.00%
Water (containing water-soluble additives)	9.00%	9.00%	9.00%

Table S2 Main fatty acids and Vitamin E content in walnut oil

Fatty acid composition		Content	Detection method
Palmitic acid	C16:0	6.77%	GB 5009.168—2016 National food safety standard - Determination of fat in foods
Palmitoleic acid	C16:1	0.13%	
Stearic acid	C18:0	1.81%	
Oleic acid	C18:1	18.02%	
Linoleic acid	C18:2	64.35%	
a-Linolenic acid	C18:3	8.14%	
Arachidic acid	C20:0	0.07%	
Arachidonic acid	C20:1	0.3%	
Vitamin E（α-TE equivalents ）	mg/100g	2.62	GB 5009.82—2016 National food safety standard — Determination of vitamins A, D and E in foods

Table S3 Main nutritional components added to feed via water and oil

Additives in water/aqueous phase	Normal	Deficiency	Supplement
L-Cystine	0.30%	0.30%	0.30%
Compound mineral mix S10022G	3.50%	3.50%	3.50%
Vitamin mixture (Vitamin E deficient)	1.00%	1.00%	1.00%
Choline bitartrate	0.25%	0.25%	0.25%
Pigment	0.002%	0.002%	0.002%
d-α-ocopheryl acetate	0.00782%	0.00000%	0.03183%
α-Tocopherol equivalents (α-TE) in walnut oil (mg/100g)	2.62	2.62	2.62
**Theoretical vitamin E content in feed**	**0.0080%**	**0.0002%**	**0.0320%**

Table S4 Primers sequences used for quantitative PCR analysis of gene expression

Primer	Forward sequence (5' to 3')	Reverse sequence (5' to 3')
RPL-19	GAAGGTCAAAGGGAATGTGTTCA	CCTTGTCTGCCTTCAGCTTGT
Arntl	TCAAGACGACATAGGACACCT	GGACATTGGCTAAAACAACAGTG
Per2	GAAAGCTGTCACCACCATAGAA	AACTCGCACTTCCTTTTCAGG
Clock	ATGGTGTTTACCGTAAGCTGTAG	CTCGCGTTACCAGGAAGCAT
Bhlhe40	ACGGAGACCTGTCAGGGATG	GGCAGTTTGTAAGTTTCCTTGC
Rorc	GACCCACACCTCACAAATTGA	AGTAGGCCACATTACACTGCT
Scd1	TTCTTGCGATACACTCTGGTGC	CGGGATTGAATGTTCTTGTCGT
Elovl3	TTCTCACGCGGGTTAAAAATGG	GAGCAACAGATAGACGACCAC
Chka1	AGCCTCGGAAAGTGCTCTTG	GTCGGCCTTGGGGAAAGATG
Chka2	GGGTGGTCTCAGTAACATGCT	GAACCCTGGACTCACCATCTT
Plin4	GTGTCCACCAACTCACAGATG	GGACCATTCCTTTTGCAGCAT
Elovl6	GAAAAGCAGTTCAACGAGAACG	AGATGCCGACCACCAAAGATA
Lpin1	CTCCGCTCCCGAGAGAAAG	TCATGTGCAAATCCACGGACT
Phospho1	ATGAGCGGGTGTTTTCCAG	ATCGAAGTCGAAGGTGAGGAG
Cyp4a14	TTTAGCCCTACAAGGTACTTGGA	GCAGCCACTGCCTTCGTAA
Cyp4a10	TTCCCTGATGGACGCTCTTTA	GCAAACCTGGAAGGGTCAAAC
Cdkn1a	CCTGGTGATGTCCGACCTG	CCATGAGCGCATCGCAATC
Cdc20	TTCGTGTTCGAGAGCGATTTG	ACCTTGGAACTAGATTTGCCAG
Gadd45a	CCGAAAGGATGGACACGGTG	TTATCGGGGTCTACGTTGAGC

Tables S1: Main nutritional components of mouse feed; Tables S2: Main fatty acids and Vitamin E content in walnut oil; Tables S3: Main nutritional components added to feed via water and oil;Tables S4: Primers sequences used for quantitative PCR analysis of gene expression.
